# Efficacy and Safety of Dextrose Prolotherapy Versus Corticosteroid Injections in Plantar Fasciitis: A Systematic Review and Meta‐Analysis

**DOI:** 10.1002/jfa2.70135

**Published:** 2026-02-17

**Authors:** Ruaa Mustafa Qafesha, Hammam Anas Ishreiteh, Adli Luay Nassourah, Omar Islam Tawil, Doaa Mashaly

**Affiliations:** ^1^ Department of Human Anatomy and Embryology Faculty of Medicine Al‐Quds University Jerusalem Palestine; ^2^ Medical Research Group of Egypt Negida Academy Arlington Massachusetts USA; ^3^ Faculty of Medicine Al‐Quds University Jerusalem Palestine; ^4^ Faculty of Medicine October 6 University 6th of October City Egypt

**Keywords:** corticosteroid injection, dextrose prolotherapy, foot function, meta‐analysis, pain, plantar fascia thickness, plantar fasciitis

## Abstract

**Background:**

Plantar fasciitis (PF) is a common cause of heel pain that affects the health‐related quality of life of many individuals and has various treatment options. Two effective interventions are corticosteroid (CS) injections and dextrose prolotherapy (DP). This study aimed to compare the efficacy and safety of DP and CS in patients with PF systematically.

**Methods:**

Relevant studies, including those comparing DP and CS for treating PF, were identified by searching electronic databases until August 2025. The visual analog scale (VAS) pain score, foot function index (FFI), and plantar fascia thickness (PFT) were compared between the groups in the short term (0.5–1 month) and mid‐term (3 months). Statistical analyses were performed via RevMan 4.5.1, and *p* < 0.05 was considered statistically significant.

**Results:**

Five RCTs and two cohort studies, with a total of 567 patients, were included in the meta‐analysis. The analysis revealed that at the short‐term follow‐up (1 month), corticosteroid injections were more effective at reducing the VAS pain scores than dextrose prolotherapy for general VAS score (MD = 1.85, 95% CI [0.05, 3.64], *p* = 0.04), the VAS score at the first step in the morning (MD = 1.26, 95% CI [0.49, 2.02], *p* = 0.001), and the VAS score for pain while walking (MD = 1.85, 95% CI [0.68, 3.02], *p* = 0.002). Similarly, at the short‐term follow‐up (1 month), the analysis revealed a significantly greater reduction in the FFI score (MD = 18.81, 95% CI [0.06, 37.55]) and PFT (MD = 0.26 mm, 95% CI [0.07, 0.45]) in the CS group than in the DP group. At 3 months, the analysis revealed a significant decrease in the FFI score (*p* = 0.003) in the DP group compared with the CS group, whereas no significant difference was observed in the VAS scores or PFT.

**Conclusion:**

In patients with plantar fasciitis, CS injections had greater efficacy than DP did in the short term; however, their efficacy became similar in the mid‐term follow‐up, with DP outperforming CS in terms of foot function. Further trials with standardized protocols and long‐term follow‐ups are needed to address potential biases.

## 1 Introduction

Plantar fasciitis (PF) is a common condition that affects the deep plantar fascia in adults and is the leading cause of heel pain [1]. It is also described in the literature as plantar heel pain, plantar fasciopathy, and plantar fasciosis, reflecting the range of tissues that may contribute to symptoms [1]. It affects up to 10% of the population during their lifetime and accounts for approximately 15% of all foot injuries [2, 3]. Most cases occur in adults aged 40–60 years and can significantly impair mobility and health‐related quality of life [4]. It is characterized by sharp heel pain during the first steps in the morning or after rest, which often improves with movement but worsens with prolonged standing. The pathophysiology of plantar heel pain is multifactorial. Although microtears and degenerative changes of the plantar fascia at its medial calcaneal insertion are well‐recognized features, other tissues may also contribute to symptoms, including the calcaneal fat pad and adjacent osseous or soft‐tissue structures. Evidence indicates that both inflammatory and degenerative pathways can occur, with local mechanical overload, altered fascia cell responses, and chronic extracellular‐matrix changes all playing a role [5]. These pathological processes may be influenced by contributing risk factors, including increased body mass index, sedentary lifestyle, and repetitive impact activities, and structural factors, such as foot deformities (e.g., pes cavus and pes planus) and leg length discrepancy [6].

The American College of Foot and Ankle Surgeons (ACFAS) Clinical Practice Guideline for heel pain recommends initial conservative management, including stretching exercises, orthoses, weight management, rest and activity modification and nonsteroidal anti‐inflammatory drugs (NSAIDs) [7, 8, 9]. For patients who fail to respond to these measures, corticosteroid (CS) injections may be considered, as they promote immediate relief for patients through their anti‐inflammatory mechanism that targets what is believed to be the inflammatory degenerative etiopathogenesis of the condition [10]. Previous studies demonstrated moderate to excellent short‐term relief; however, the guidelines caution against repeated injections due to the risk of complications such as plantar fascia rupture and heel fat pad atrophy [9], which limits their long‐term use owing to the associated morbidity [11].

Prolotherapy, typically hyperosmolar dextrose, has emerged as a promising regenerative treatment that promotes tissue regeneration and repair through the induction of a controlled local inflammatory response [12]. This stimulates fibroblast proliferation, collagen deposition, and vascular growth [13, 14, 15]. Recent research has shown promising results in the treatment of PF using prolotherapy, as it offers similar pain and functional improvements to platelet‐rich plasma (PRP) and extracorporeal shockwave therapy (ESWT) [16], with ESWT sometimes achieving faster early relief and PRP showing marginal advantages at certain time points [16, 17]. However, prolotherapy often results in similar long‐term outcomes while being less invasive, more affordable, and technically straightforward when compared to PRP and ESW [18, 19, 20].

Although other reviews have compared CS or prolotherapy injections with other treatments, such as ESWT and PRP, to date, no meta‐analysis has comprehensively compared dextrose prolotherapy (DP) and CS in PF using the current body of evidence. Prior reviews attempted this comparison but included only two eligible trials [18, 19, 20]. Since then, multiple randomized controlled trials (RCTs) and well‐designed observational studies have been published, enabling a more comprehensive and up‐to‐date evaluation [17, 21, 22, 23, 24, 25, 26]. This systematic review and meta‐analysis synthesized both randomized and observational data to compare the effectiveness and safety of DP versus CS in PF, with pain intensity and functional improvement as primary outcomes and plantar fascia thickness (PFT) and adverse events as secondary outcomes, to provide updated, evidence‐based guidance for clinicians.

## 2 Methods

This systematic review was conducted according to the Preferred Reporting Items for Systematic Reviews and Meta‐Analyses (PRISMA), and the protocol is registered in PROSPERO (CRD420251126876).

### 2.1 Information Sources and Search Strategy

We systematically searched PubMed, the Cochrane Central Register of Controlled Trials, Web of Science, Scopus, and Google Scholar from inception to 31 October 2025. The search strategy was developed according to the PICOS framework and incorporated a broad range of terms related to plantar fasciitis (e.g., “plantar heel pain,” “plantar fasciopathy,” “plantar fasciosis”), dextrose prolotherapy (e.g., “dextrose injection,” “hypertonic dextrose”), and corticosteroid injections (e.g., “glucocorticoid,” “triamcinolone”).

The term “plantar fasciitis” was retained as a core keyword because it remains the terminology consistently used in controlled trials evaluating dextrose prolotherapy and corticosteroid injections, ensuring alignment with the intervention literature.

The search strategy was adapted for each database without the use of filters or language restrictions. Full reproducible search strings for all databases are provided in Supporting Information S1: Table S1.

### 2.2 Inclusion and Exclusion Criteria

Eligible studies included randomized controlled trials (blinded or unblinded) and high‐quality prospective or retrospective cohort studies directly comparing dextrose prolotherapy (DP) with corticosteroid (CS) injections in adults (≥ 18 years) diagnosed with plantar fasciitis. Both study designs were included because several outcomes had limited RCT data, and excluding cohort studies would have removed important clinically relevant outcomes from the analysis. Studies were required to report at least one of the following clinical outcomes: (1) pain intensity measured via the visual analog scale (VAS) or numerical rating scale (NRS); (2) functional improvement assessed via the Foot Function Index (FFI); (3) plantar fascia thickness as evaluated via ultrasound; or (4) the incidence of treatment‐related adverse events or complications. The included interventions consisted of either ultrasound‐guided or palpation‐guided injections of dextrose prolotherapy, with corticosteroid injections serving as the comparator.

The exclusion criteria were as follows: case reports, case series, narrative reviews, abstracts without full‐text availability, book chapters, duplicate publications, and studies lacking direct comparisons between the two treatment modalities or failing to report separate outcome data for each group. Additionally, studies involving participants with coexisting foot or ankle pathologies (e.g., rheumatoid arthritis, tarsal tunnel syndrome, or Achilles tendinopathy) were excluded to avoid confounding the plantar fasciitis‐specific outcomes.

### 2.3 Data Extraction Process and Quality Assessment

Two reviewers (H.A.I. and D.M.) independently and blindly screened titles/abstracts and full texts for eligibility. Disagreements at the study selection stage were resolved through discussion, and when consensus could not be reached, by consultation with a third reviewer (R.M.Q). The data were extracted by two reviewers (H.A.I, A.L.N) using a predefined extraction sheet. The extracted information included author, publication year, country, center, study design, inclusion criteria, Sample size, sex distribution, mean age, and detailed intervention descriptions (dextrose prolotherapy and corticosteroid injections), such as injection type, frequency, and guidance (ultrasound or palpation). Outcome data were extracted using standardized time points: baseline and posttreatment measurements were extracted at 1 month and 3 months; if 1‐month data were unavailable, 2‐week values were used instead. Then, change‐from‐baseline values were calculated for both short‐term (0.5–1 month) and mid‐term follow‐up (3 months). Adverse events were also recorded. For studies with multiple intervention groups, only data from the DP and CS arms were extracted. All data were exported into a Microsoft Excel spreadsheet. Disagreements during data extraction were resolved through discussion and, when necessary, by consultation with a third reviewer (R.M.Q).

For quality assessment, two independent reviewers (H.A.I and A.L.N) evaluated the risk of bias via the Cochrane RoB 2 tool for randomized controlled trials (RCTs), which examines five domains: bias arising from the randomization process, deviations from intended interventions, missing outcome data, measurement of outcomes, and selection of the reported results. Additionally, the Newcastle‒Ottawa Scale (NOS) was employed to assess the selection, comparability, and outcome domains for cohort studies, with scores of ≥ 7 indicating high quality. Discrepancies in risk of bias or quality assessment were resolved through discussion and, if consensus could not be reached, by consultation with a third reviewer (R.M.Q. and D.M.).

### 2.4 Outcome Definitions

Pain intensity was assessed via the visual analog scale (VAS) [27], a validated scale ranging from 0 (no pain) to 10 (worst pain). If reported on a 0–100 scale, values were converted to 0–10 for consistency. Higher scores indicate greater pain intensity. Functional status was measured via the Foot Function Index (FFI) [28], a 23‐item questionnaire scored from 0 to 100, with higher scores indicating worse function. When reported as a raw cumulative score out of 230, values were converted to a 0–100 scale via proportional transformation. A decrease in the FFI score indicates functional improvement. Plantar fascia thickness (PFT) was assessed via ultrasound at the calcaneal insertion; greater thickness is associated with more severe disease, and a reduction reflects structural improvement. All PFT values were extracted in millimeters without conversion.

### 2.5 Statistical Analysis and Assessment of Heterogeneity

A narrative synthesis of the included studies was conducted, structured by intervention type, participant characteristics, outcome measures, and follow‐up periods. Meta‐analyses were performed where at least two studies reported the same outcome at the same time point. All outcomes were analyzed as change‐from‐baseline values at both short‐term (0.5–1 month) and mid‐term (3 months) follow‐up. Pooled estimates were calculated via mean differences (MDs) with 95% confidence intervals (CIs). Standardized mean differences (SMDs) were not used, as all outcomes were standardized to the same scale before pooling. Heterogeneity was first assessed using the χ^2^ *p*‐value (with *p* < 0.10 indicating significant heterogeneity), and the degree of heterogeneity was then quantified using the *I* ^2^ statistic. A fixed‐effect model was applied when no significant heterogeneity was detected; otherwise, a random‐effects model was used. Subgroup analyses were conducted only for the injection guidance method (ultrasound vs. palpation) when sufficient studies were available for an outcome. Sensitivity analysis was performed via the leave‐one‐out (jackknife) method to evaluate the influence of individual studies on pooled results. All analyses were performed via RevMan 5.4.1, and *p* values < 0.05 were considered statistically significant. In line with Cochrane guidance on the assessment of publication bias in small meta‐analyses, publication bias was evaluated using Egger's weighted regression test implemented in the metafor package in R. Owing to the small number of studies per outcome, the results were considered exploratory and interpreted with caution [29].

## 3 Results

### 3.1 Literature Search and Summary of the Included Studies

**FIGURE 1:**
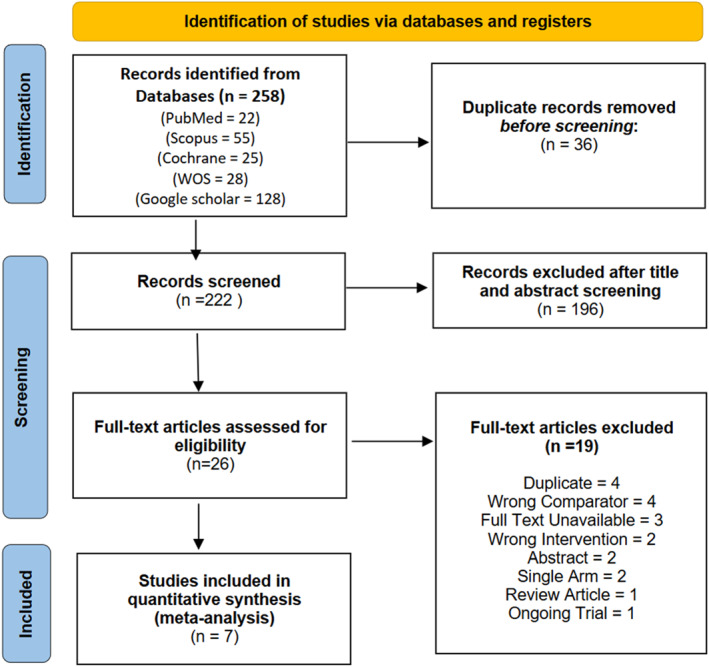
The study selection process followed PRISMA guidelines.

As illustrated in Figure 1, the search across the five electronic databases yielded 216 records. After 31 duplicates identified via EndNote were removed, 185 records remained for title and abstract screening. Of these, 26 articles underwent full‐text assessment for eligibility, and ultimately, seven studies met the inclusion criteria and were included in the quantitative synthesis (meta‐analysis). A complete list of full‐text exclusions (*n* = 19), together with citation details and reasons for exclusion, is provided in Supporting Information S1: Table S2 [30].

The seven studies [17, 21, 22, 23, 24, 25, 26] included in this review were published between 2018 and 2025. Among them, five were randomized controlled trials, and two were observational cohort studies. All studies directly compared DP and CS injections for the treatment of PF. The corticosteroids administered included methylprednisolone acetate (40 mg/mL) or triamcinolone, which were used alone or in combination with local anesthetics, such as lidocaine or prilocaine. Dextrose concentrations ranged from 5% to 50% and were diluted with local anesthetics. Injection guidance varied: four studies employed ultrasound guidance, and three used palpation‐guided techniques. Six studies assessed outcomes up to 3 months post‐injection, whereas one study extended the follow‐up to 36 months, providing long‐term data on treatment durability. Table 1 provides more details regarding the included studies. The seven studies included a total of 567 participants with mean ages ranging from approximately 42–54 years and a predominance of female participants (up to 88% in one study). The mean BMI across studies was between 27.7 and 32.5 kg/m^2^, indicating that most of the participants were overweight or obese. All studies included participants with symptom durations of more than 6 weeks prior to enrollment. Baseline characteristics of the included studies are summarized in Table 2. Table 3 presents the extracted mean and standard deviation values, encompassing baseline assessments and post‐injection outcomes at short‐term and mid‐term follow‐up.

**TABLE 1:** Summary and characteristics of the included study in the meta‐analysis.

Study ID	Study design/blinding status	Country/centers	Inclusion criteria	Sample size, *N* (DP/CS)	Injection type		Injection frequency/guidance	FU period	Conclusion	Funding
					DP	CS				
Teymouri 2025	Double‐blind RCT (participants + care providers)	Iran, Single Center	Adult patients ≥ 18 years with a definitive PF diagnosis confirmed by US (plantar fascia thickness ≥ 4 mm) at the physical medicine and rehabilitation clinic, unilateral foot symptoms recalcitrant to conservative therapy, such as weight reduction, physical therapy, and night splints, had to be present for at least 6 months	38 (19/19)	1 mL of dextrose 50% solution + 1 mL of lidocaine 2%	40 mg of methylprednisolone acetate + 1 mL of lidocaine 2%	DP: 1x	12 weeks	Both CS and DP were effective in treating PF by reducing pain and improving foot function index in the middle term. Although CS ensures better short‐term outcomes, its effectiveness tends to wane over time. In contrast, DP is more effective in the middle term	None/declared
							CS: 1x			
							US‐guided			
Puranik 2025	Comparative observational study	India, Single Center	1. Individuals ≥ 18 years with PF. 2. Diagnosis was made based on the ICD criteria: a. Pain in the plantar medial heel region upon palpation. b. Pain that worsened after inactivity and with prolonged weight‐bearing. c. Pain triggered by increased weight‐bearing activities	50 (25/25)	2 mL of 25% dextrose injections	40 mg of triamcinolone acetonide mixed with 1 mL of normal saline	DP: 1x	12 weeks	Both CS and DP injections are effective in managing PF. Although steroid provides faster symptom relief, dextrose offers sustained benefits with fewer complications, making it a promising alternative for long‐term management	None/declared
							CS: 1x			
							Palpation‐guided			
Mousa 2025	RCT/NR	Egypt, NA	1. Cases ≥ 18 years with heel pain for more than 3 months. 2. Failure to respond to conservative treatment, including NSAIDs, physiotherapy, and stretching exercises. 3. Bilateral PF patients were evaluated, focusing on the side exhibiting more pronounced symptoms	75 (3 groups), (25/25)	3.6 mL 25% dextrose + 0.4 mL lidocaine	40 mg/mL methylprednisolone acetate + 2 mL of 2% prilocaine	DP: 3x	12 weeks	ESWT, CS injection, and DP reduce chronic PF pain and fasciopathy thickness. Short‐term and unfavorable effects of CS injections make ESWT and DP better over time	None/declared
							CS:1x			
							Palpation‐guided			
Karakılı 2023	Double‐blind RCT (participants + outcome assessors)	Turkiye Multicenter,2 centers	18–65 years old, heel pain of more than 3 months, worsening of plantar fascia tenderness by manual compression of the medial border of the calcaneus, proximal PFT greater than 4 mm and areas of hypoechogenicity, history of unsuccessful conservative treatments	146 (3 groups), (44/42)	3.6 mL 30% dextrose solution + 0.4 mL lidocaine	Methylprednisolone acetate 40 mg/1 mL + 2% prilocaine	DP: 2x	12 weeks	DP, phonophoresis, and CS injection are beneficial as safe treatment modalities in the early period of PF treatment. The improvement of HSI and SF‐36 GH subscale lasts longer with DP, but US findings do not change in the 3rd month of these treatment applications	None reported
							CS:1x			
							US‐guided			
Calısal 2022	Retrospective observational/‐	Turkiye, Single Center	Patients admitted in clinic between 2019 and 2020 for heel pain and clinically and radiographically diagnosed as PF were recruited in the study	60, (30/30)	2 mL 15% dextrose + 2 mL 2% prilocaine	2 mL 2% prilocaine + 2 mL 40 mg methylprednisolone	DP: 2x	3 months	DP and CS injection treatments provide significant functional outcomes in short‐term follow‐up of the treatment of PF. CS injection results in superior clinical healing than prolotherapy	None
							CS: 1x			
							Palpation‐guided			
Raissi 2021	RCT/NR	Iran, NA	Patients between 18 and 75 years with a diagnosis of chronic PF based on clinical symptoms (NRS score > 4 for more than 8 weeks), signs, and US findings (proximal plantar fascia thickness greater than 4 mm and areas of hypo‐echogenicity)	40, (20/20)	2 mL of 20% dextrose + 1 mL of 1% lidocaine hydrochloride	1 mL of 40 mg methylprednisolone + 1 mL normal saline (0.9% sodium chloride) + 1 mL of 1% lidocaine hydrochloride	DP: 1x	12 weeks	Both methods are effective. Compared with DP, and our results show that CS injection may have superior therapeutic effects early	Funded by Iran University of Medical Sciences
							CS: 1x			
							US‐guided			
Uğurlar 2018	RCT/NR	Turkiye, Multicenter	Age ≥ 18 years with pain on palpation of plantar medial calcaneal tubercle for ≥ 6 months, BMI < 30 kg/m^2^, VAS for pain intensity > 5 for participant's self‐assessment of pain on first few minutes of walking in morning, pain worse on waking up in the morning or after a period of rest. Heel spur on lateral radiograph of the foot, failure to respond to treatment modalities	158 (4 groups), (40/40)	1 mL of bupivacaine 5 mg/mL + 3 mL of 5% dextrose, + 6 mL of 0.9% physiologic sodium chloride solution	1 mL of betamethasone 40 mg/mL + 2 mL bupivacaine 5 mg/mL	DP: 3X	36 months	The CS injection was more effective in the first 3 months and ECSW shock wave therapy was an effective treatment method in the first 6 months in regard to pain. The CS injection lost its effectiveness during the follow‐up period. The effect of DP and platelet rich plasma was seen within 3–12 months; however, at the 36‐month follow‐up point, no differences were found among the four treatments	None reported
							CS: 3X			
							US‐guided			

Abbreviations: CS, corticosteroids; DP, dextrose prolotherapy; FFI, foot function index; FU, follow up; PF, plantar fasciitis; RCT, randomized controlled study; US, ultrasound, VAS, visual analog scale; y, year.

**TABLE 2:** The baseline characteristics of the included studies.

Study ID	Number of participants		F, *N* (%)		Age (Y) mean (SD)		BMI (kg/m^2^) (SD)		Baseline VAS (SD)		Baseline FFI (SD)	
	DP	CS	DP	CS	DP	CS	DP	CS	DP	CS	DP	CS
Teymouri 2025	19	19	13 (68.5)	11 (58)	48.8 (5.9)	48.2 (6.2)	27.73 (2.3)	27.75 (2.27)	7.73 (1.28)	7.89 (1.19)	76.42 (7.29)	75.05 (7.36)
Puranik 2025	25	25	17 (68)	15 (60)	44.36 (8.8)	44.36 (8.8)			Walking VAS 8.92 (1.03)	Walking VAS 8.48 (1.15)	NR	NR
									Morning VAS 9.44 (0.71)	Morning VAS 9.32 (0.85)		
Mousa 2025	25	25	20 (80)	22 (88)	42.9 (9.83)	46.8 (8.8)	31.1 (3.42)	32.5 (3.23)	7.76 (1.16)	7.88 (1.3)	154.5 (20.7)	161 (15.3)
Karakılı 2023	44	42	NR	NR					70.60 (11.85)	71.40 (11.06)	61.78 (9.13)	61.65 (10.15)
Calısal 2022	30	30	18 (60)	16 (53.3)	54.13 (9.38)	47.46 (6.74)	31.57 (4.58)	32.02 (4.89)	8.03 (1.09)	7.76 (0.93)	176.1 (16.9)	181 (13.9)
Raissi 2021	20	20	15 (75)	18 (90)	50.3 (11.64)	42.15 (9.42)	27.4 (3.6)	28.56 (4.41)	Morning VAS 7.15 (1.63)	Morning VAS 6.95 (2.06)	NR	NR
									Walking VAS 5.55 (1.05)	Walking VAS 5.15 (1.13)		
Uğurlar 2018	40	40	19 (47.5)	23 (57.5)	37.5 (25–62)^a^	40.1 (21–56)^a^	26.7 (22.2–29.7)^a^	27.3 (21.5–29.3)^a^	7.0 (6.4)	7.4 (5.5)	140.3 (128.7)	146.5 (133.4)

Abbreviations: BMI, body mass index; CS, corticosteroids; DP, dextrose prolotherapy; F, female; FFI, foot function index; *N*, number; NR, not reported; SD, standard deviation; VAS, visual analog scale; Y, year.

^a^ Data presented as the mean (range).

**TABLE 3:** Baseline and posttreatment outcomes in patients with PF.

Study ID	Group	Duration	VAS mean, (SD)	FFI mean, (SD)	PFT mean, (SD)
Uğurlar 2018	DP	Baseline	7.0 (6.4)	140.3 (128.7)	N/A
		1 M	6.9 (6.5)	138.6 (125.6)	N/A
		3 M	2.8 (2.3)	81.4 (72.4)	N/A
	CS	Baseline	7.4 ± 5.5	146.5 ± 133.4	N/A
		1 M	3.2 ± 2.4	90.1 ± 77.3	N/A
		3 M	4.4 ± 3.5	107.9 ± 97.6	N/A
Raissi 2021	DP	Baseline	7.15 (1.63)	N/A	3.76 (0.36)
		2 W	4.65 (1.81)	N/A	4.29 (0.33)
		12 W	2.7 (1.65)	N/A	3.9 (0.25)
	CS	Baseline	6.95 (2.06)	N/A	3.56 (0.38)
		2 W	2.75 (2.67)	N/A	3.89 (0.47)
		12 W	2.65 (3.03)	N/A	3.86 (0.48)
Calısal 2022	DP	Baseline	8.03 (1.09)	176.1 (16.9)	N/A
		3 M	4.93 (1.11)	126.9 (7.4)	N/A
		12 M	N/A	N/A	N/A
	CS	Baseline	7.76 (0.93)	181 (13.9)	N/A
		3 M	4.23 (0.62)	121.1 (16.1)	N/A
		12 M	N/A	N/A	N/A
Karakılı 2023	DP	Baseline	70.60 (11.85)	61.78 (9.13)	5.45 (1.04)
		1 M	27.20 (23.82)	26.97 (20.70)	3.43 (1.33)
		3 M	30.45 (27.86)	27.91 (21.77)	3.53 (9.41)
	CS	Baseline	71.40 (11.06)	61.65 (10.15)	5.31 (1.07)
		1 M	27.20 (26.57)	25.85 (23.56)	3.22 (1.24)
		3 M	30.65 (27.35)	35.69 (24.77)	3.74 (1.36)
Mousa 2025	DP	Baseline	7.76 (1.16)	154.5 (20.7)	5.29 (0.61)
		1 M	5.72 (1.24)	125.8 (20)	4.95 (0.57)
		3 M	2.92 (1.12)	102.2 (17.5)	4.38 (0.52)
	CS	Baseline	7.88 (1.3)	161 (15.3)	5.34 (1.07)
		1 M	3.96 (0.98)	115.6 (17.3)	4.46 (0.64)
		3 M	2.24 (1.13)	110.6 (19)	4.27 (0.54)
Teymouri 2025	DP	Baseline	7.73 (1.28)	76.42 (7.29)	N/A
		1 M	7.15 (1.21)	70.70 (6.12)	N/A
		3 M	3.21 (1.03)	35 (6.12)	N/A
	CS	Baseline	7.89 (1.19)	75.05 (7.36)	N/A
		1 M	3.78 (0.19)	35 (5.7)	N/A
		3 M	4.63 (1.11)	39 (5.9)	N/A
Puranik 2025	DP	Baseline	7.73 (1.28)	76.42 (7.29)	N/A
		1 M	7.15 (1.21)	70.70 (6.12)	N/A
		3 M	3.21 (1.03)	35 (6.12)	N/A
	CS	Baseline	7.89 (1.19)	75.05 (7.36)	N/A
		1 M	3.78 (0.19)	35 (5.7)	N/A
		3 M	4.63 (1.11)	39 (5.9)	N/A

Abbreviations: CS, corticosteroids; DP, dextrose prolotherapy; FFI, foot function index; N/A, not applicable; PF, plantar fasciitis; PFT, plantar fascia thickness; SD, standard deviation; VAS, visual analog scale.

### 3.2 Quality of the Included Studies

**FIGURE 2:**
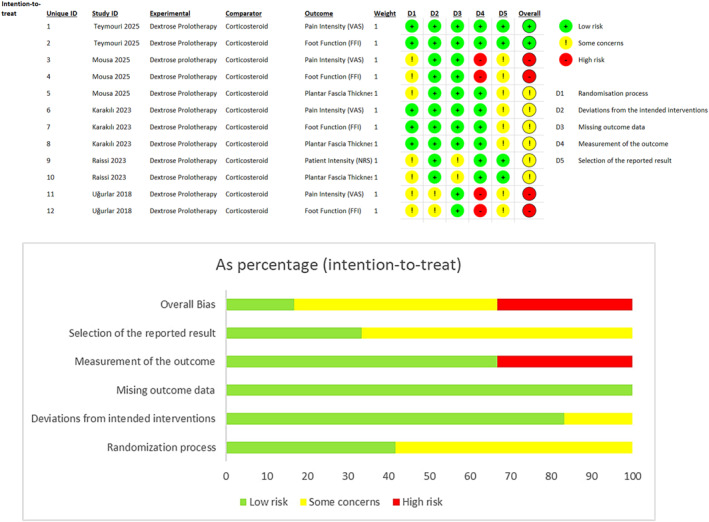
Risk of bias assessment summary for included randomized controlled trials.

Using the RoB‐2 tool at the outcome level, 12 outcomes across five RCTs were assessed (Figure 2). In Teymouri 2025 [23] and Karakılıç 2023 [26], pain intensity (VAS) and the Foot Function Index (FFI) were generally rated at low risk of bias across the domains related to the randomization process, deviations from intended interventions, missing outcome data, and outcome measurement (domains 1–4). For the domain addressing selection of the reported result (domain 5), Teymouri [23] remained low risk, whereas Karakılıç [26] was judged to have some concerns due to lack of a pre‐specified analysis plan. Karakılıç 2023 [26] also assessed PFT, which received an overall rating of some concerns, primarily because of issues in the selection of the reported result (domain 5).

In Raissi 2021 [25], both pain intensity (NRS) and PFT were judged as having some concerns, influenced mainly by missing outcome data (four participants not included in the analysis) and uncertainty regarding the selection of the reported result (domain 5) due to the absence of an accessible protocol.

Higher risk of bias was identified for outcomes in Mousa 2025 [24] and Uğurlar 2018 [17]. Across both trials, VAS and FFI were rated at high risk, largely due to issues in outcome measurement, specifically, unblinded assessors who could have been influenced by knowledge of treatment allocation (domain 4). In addition, both studies lacked a clear pre‐specified analysis plan, raising concerns about the selection of the reported result (domain 5), which further contributed to the high‐risk judgments.

The two observational studies evaluated via the Newcastle–Ottawa Scale both received a total score of 7, indicating good methodological quality with satisfactory selection, comparability, and outcome assessment (see Supporting Information S1: Table S3).

### 3.3 Efficacy Outcomes

#### 3.3.1 Pain Intensity (VAS Score) Outcome

Although all studies used the same VAS scale to assess pain intensity, they differed in the context of measurement. Four studies reported a general VAS score, reflecting overall pain severity without reference to activity or time of day, whereas other trials measured the VAS score for the first step (morning pain) or pain while walking. To address this variation, separate subgroup analyses were conducted for the general VAS, first‐step VAS, and walking VAS scores.

##### 3.3.1.1 General VAS

**FIGURE 3:**
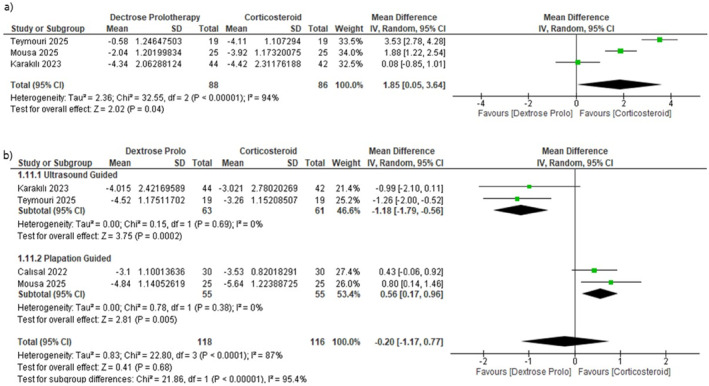
Forest plot for comparison between CS and DP regarding (a) the general VAS score change at 1 month. (b) General VAS score change at 3 months.

Three RCTs (*n* = 178) evaluated general VAS scores at short‐term follow‐up (1 month). The pooled analysis demonstrated a statistically significant greater reduction in pain in the CS group than in the DP group (MD = 1.85, 95% CI [0.05, 3.64], *p* = 0.04) (Figure 3a). Considerable heterogeneity was found (*p* < 0.00001, *I* ^2^ = 94%) and was not resolved through sensitivity analysis.

In the mid‐term (3 months), no significant difference was observed between CS and DP (MD = −0.20, 95% CI –1.17 to 0.77, *p* = 0.68), with substantial heterogeneity (*p* < 0.0001, *I* ^2^ = 87%) (Figure 3b). An RCT‐only sensitivity analysis produced the same conclusion (MD = −0.46, 95% CI –1.89 to 0.97, *p* = 0.53), although heterogeneity remained high (*p* < 0.0001, *I* ^2^ = 89%) (Supporting Information S1: Figure S1).

Subgroup analysis via the injection guidance method resolved heterogeneity and revealed that the relative effectiveness of the two treatments differed significantly between subgroups (test for subgroup differences *p* < 0.001). In ultrasound‐guided studies (2 studies; *n* = 124), DP produced greater pain reduction than did CS (MD = −1.18, 95% CI [–1.79 to −0.56], *I* ^2^ = 0%), whereas in palpation‐guided studies (2 studies; *n* = 110), CS was favored (MD = 0.56, 95% CI [0.17 to 0.96], *I* ^2^ = 0%). This statistically significant difference between subgroups indicates that the injection guidance method may affect the comparative effectiveness of DP and CS (Table 4).

**TABLE 4:** Summary of our analysis results.

Analysis		MD and 95%CI	*p* value	Heterogeneity		Number of studies	Conclusion
				*p* value	*I* ^2^		
General VAS	Short term	1.85, 95% CI [0.05, 3.64]	*p* = 0.04	*p* < 0.00001	*I* ^2^ = 94%	3	Greater pain reduction in CSI group compared with the DP group
General VAS	Mid‐term	−0.20, 95% CI [−1.17, 0.77]	*p* = 0.68	*p* < 0.0001	*I* ^2^ = 87%	3	No significant difference between CSI and DP
General VAS mid‐term according to US guidance	US‐guided	−1.18, 95% CI [−1.79, −0.56]	*p* = 0.0002	*p* = 0.69	*I* ^2^ = 0%	2	DP produced greater pain reduction than CSI
	Palpation‐guided	0.56, 95% CI [0.17, 0.96]	*p* = 0.005	*p* = 0.38	*I* ^2^ = 0%	2	CSI produced greater pain reduction than DP
Morning VAS	Short term	1.26, 95% CI [0.49, 2.02]	*p* = 0.001	*p* = 0.77	*I* ^2^ = 0%	3	Greater pain reduction in the CSI group compared with the DP group
	Mid‐term	0.10, 95% CI [−0.29, 0.50]	*p* = 0.61	*p* = 0.77	*I* ^2^ = 0%	3	No significant difference between CSI and DP
Walking VAS	Short term	1.85, 95% CI [0.68, 3.02]	*p* = 0.002	*p* = 0.05	*I* ^2^ = 74%	2	Greater reduction in the CSI group compared with the DP group
	Mid‐term	−0.44, 95% CI [−0.96, 0.08]	*p* = 0.10	*p* = 0.99	*I* ^2^ = 0%	2	No significant difference between CSI and DP
FFI	Short term	18.81, 95% CI [0.06, 37.55]	*p* = 0.05	*p* < 0.00001	*I* ^2^ = 94%	4	Greater reduction in the CSI group compared with the DP group
	Mid‐term	−5.47, 95% CI [−9.03, −1.92]	*p* = 0.003	*p* = 0.83	*I* ^2^ = 0%	4	DP produced greater FFI reduction than CSI
PFT	Short term	0.26 mm, 95% CI [0.07, 0.45]	*p* = 0.007	*p* = 0.33	*I* ^2^ = 11%	3	Greater PFT reduction in the CSI group compared with the DP group
	Mid‐term	−0.11 mm, 95% CI [−0.29, 0.06]	*p* = 0.21	*p* = 0.48	*I* ^2^ = 0%	4	No significant difference between CSI and DP

##### 3.3.1.2 Morning VAS

**FIGURE 4:**
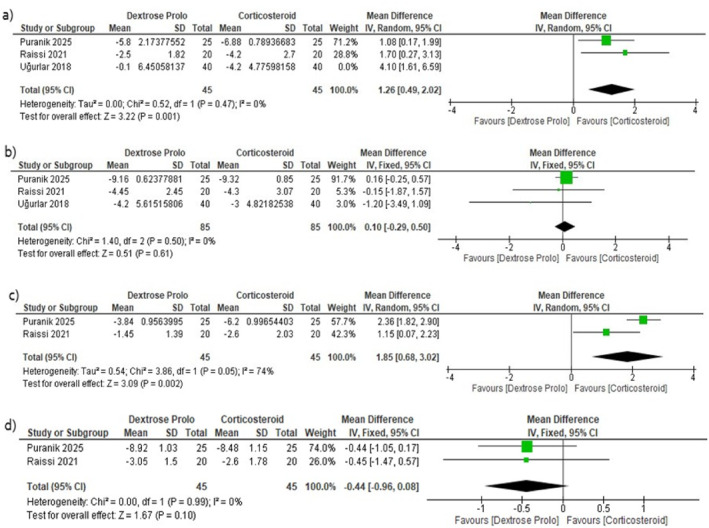
Forest plot for comparison between CS and DP regarding (a) morning VAS score change at 0.5–1 month. (b) Morning VAS score change at 3 months. (c) Walking VAS change at 0.5 months. (d) Walking VAS change at 3 months.

In the short term (1 month), the initial pooled analysis favored CS (MD = 1.89, 95% CI 0.52–3.26, *p* = 0.007), although moderate heterogeneity was present (*p* = 0.08, *I* ^2^ = 61%) (Supporting Information S1: Figure S2). After excluding Uğurlar et al., which resolved heterogeneity, the effect remained statistically significant in favor of CS (MD = 1.26, 95% CI 0.49–2.02, *p* = 0.001; *I* ^2^ = 0%) (Figure 4a) and Table 4.

An RCT‐only sensitivity analysis (excluding the cohort study) produced a consistent effect direction, again favoring CS (MD = 2.67, 95% CI 0.36–4.99, *p* = 0.02; heterogeneity *p* = 0.10, *I* ^2^ = 63%) (Supporting Information S1: Figure S3).

Conversely, at the mid‐term (3 months), there was no significant difference between groups (MD = 0.10, 95% CI –0.29 to 0.50, *p* = 0.61), with no heterogeneity (*p* = 0.50, *I* ^2^ = 0%) (Figure 4b). An RCT‐only sensitivity analysis showed the same finding (MD = −0.53, 95% CI –1.91 to 0.85, *p* = 0.45), also without heterogeneity (Supporting Information S1: Figure S4).

##### 3.3.1.3 Walking VAS

Two studies provided data on VAS scores for pain while walking. At short‐term follow‐up (2 weeks), CS was associated with a significantly greater reduction in walking pain than DP was (MD = 1.85, 95% CI [0.68, 3.02], *p* = 0.002) (Figure 4c), with substantial heterogeneity observed (*p* = 0.05, *I* ^2^ = 74%). At mid‐term (3 months), there was no significant difference between interventions (MD = −0.44, 95% CI [–0.96, 0.08], *p* = 0.10), and the data were homogeneous (*p* = 0.99, *I* ^2^ = 0%) (Figure 4d and Table 4).

#### 3.3.2 Foot Function Index (FFI)

**FIGURE 5:**
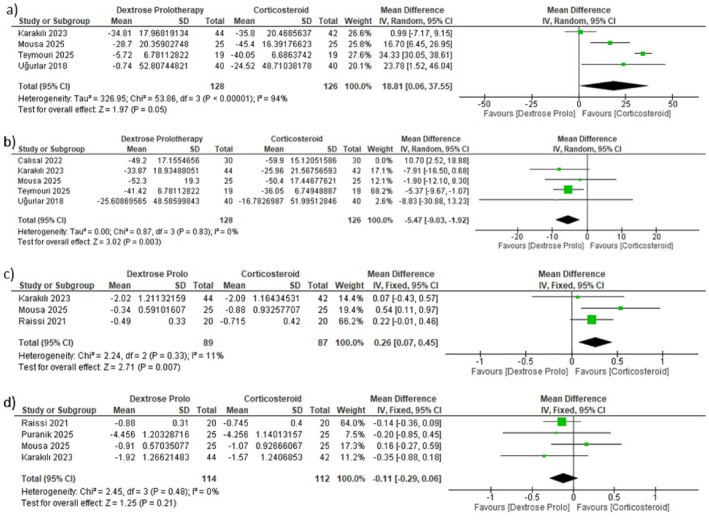
Forest plot for comparison between CS and DP regarding (a) FFI change at 1 month. (b) FFI change at 3 months, (c) PFT change at 0.5–1 month, (d) PFT change at 3 months.

Four RCTs (*n* = 254) evaluated the FFI at short‐term follow‐up (1 month). The pooled analysis demonstrated a significantly greater reduction in FFI scores in the CS group than in the DP group (MD = 18.81, 95% CI [0.06, 37.55], *p* = 0.05). Considerable heterogeneity was present and persisted despite the sensitivity analysis (*p* < 0.00001, *I* ^2^ = 94%) (Figure 5a and Table 4).

At 3 months, data from five studies (*n* = 314) revealed no statistically significant difference between CSs and DPs (MD = −1.92, 95% CI [–9.12, 5.28], *p* = 0.6) (Supporting Information S1: Figure S5). However, heterogeneity was detected (*p* = 0.009, *I* ^2^ = 70%), which was resolved when Calısal et al. was excluded, leaving a model composed exclusively of RCTs. The resulting estimate favored DP and remained statistically significant (MD = −5.47, 95% CI [–9.03, −1.92], *p* = 0.003), indicating superior foot function with DP at the 3‐month follow‐up (Figure 5b and Table 4).

#### 3.3.3 Plantar Facia Thickness (PFT)

Plantar fascia thickness was assessed sonographically, and values measured at the calcaneal insertion were used for analysis. At short‐term follow‐up (0.5–1 month), pooled analysis of three studies revealed a significantly greater reduction in PFT in the CS group than in the DP group (MD = 0.26 mm, 95% CI [0.07, 0.45], *p* = 0.007). At 3 months, pooled data from four studies showed no significant difference between groups (MD = −0.11 mm, 95% CI [–0.29, 0.06], *p* = 0.21), with homogeneous data across both time points. Figure 5c,d and Table 4. The RCT‐only model produced the same finding, also showing no significant difference (MD = −0.11 mm, 95% CI –0.29 to 0.08, *p* = 0.26) (Supporting Information S1: Figure S6).

### 3.4 Safety Outcomes

Four studies reported adverse event data. Karakılıç et al., Raissi et al., and Uğurlar et al. reported no complications in either group. Mousa et al. reported heel fat pad atrophy in six patients (25%) in the CS group and transient paresthesia at the injection site in five patients (20%) in the DP group. Owing to the small number of reporting studies and variability in adverse event reporting, a pooled meta‐analysis was not feasible.

### 3.5 Other Outcomes

Additional functional, structural, and patient‐reported outcomes were assessed in individual trials but could not be included in the meta‐analysis, as they were reported by only one study each. These outcomes included the Foot and Ankle Ability Measure (FAAM), plantar fascia echogenicity, heel sensitivity index, SF‐36 score, heel fat pad thickness, maximum walking distance, and American Foot and Ankle Scale (AFAS) score. Across these measures, both the DP and CS groups generally improved from baseline at various follow‐up points, with between‐group differences rarely reaching statistical significance. The detailed follow‐up intervals and key findings for each outcome are summarized in Table 5.

**TABLE 5:** Outcomes reported in single studies (not included in the meta‐analysis).

Outcome	Study	Follow‐up points (month)	Key findings
Foot and ankle ability measure (FAAM)	Raissi 2021	0.5 month	Both DP and CS groups showed significant improvement from baseline. CS achieved significantly higher FAAM‐S at 2 weeks
		3 months	No statistically significant difference between groups in FAAM‐A at any time point
Plantar fascia echogenicity (ultrasound)	Raissi 2021	0.5 month	Echogenicity improved in both groups from baseline at both follow‐up points
		3 months	No between group comparison for this outcome
Heel sensitivity index	Karakılıç 2023	1 month	Significant reduction from baseline in both groups at all follow‐ups
		3 months	No statistically significant difference between groups in HSI at any time point
SF‐36 (short form health survey)	Karakılıç 2023	1 month	Improvement in all SF‐36 subdomains for both groups at all follow‐ups
		3 months	No statistically significant difference between groups
Heel fat pad thickness (ultrasound)	Mousa 2025	1 month	Improvement in DP groups but not the CS group at follow‐up points
		3 months	No statistically significant difference between groups
Maximum walking distance	Puranik 2025	0.5 month, 1 month	Significant improvement in both groups at all follow‐ups compared to baseline
		1.5 month, 2 month	CS achieved statistically significant increase in walking distance more than the DP group. CS led to faster mobility gains, whereas DP achieved comparable results by 3 months
		2.5 month, 3 months	
American Foot and Ankle Score (AFAS)	Puranik 2025	0.5 month, 1 month	Both groups demonstrated marked posttreatment improvement across all follow‐up intervals
		1.5 month, 2 month,	The CS group exhibited significantly faster gains in AFAS scores, indicating a more rapid functional benefit during the early follow‐up period (0.5–2.5 months). However, by 3 months, both treatments reached similar functional outcomes with no significant difference between the two treatments
		2.5 month, 3 months	

### 3.6 Publication Bias

**FIGURE 6:**
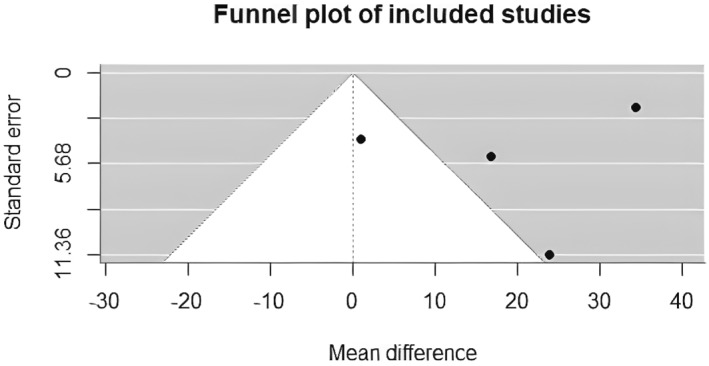
Funnel plot assessing small‐study effects for the pooled outcome. Effect size (mean difference) is plotted against standard error.

Egger's weighted regression test did not suggest evidence of small‐study effects (*p* = 0.94). Given the small number of included studies, this finding should be interpreted as exploratory, see Figure 6.

## 4 Discussion

PF is a prevalent and often persistent cause of heel pain, frequently resulting in prolonged discomfort, functional limitations, and a substantial healthcare burden. Although many patients respond to conservative measures, persistent cases often necessitate injection‐based therapies [30, 31]. CS injections remain widely used for rapid pain relief, whereas DP has attracted interest as regenerative alternatives that target long‐term structural healing.

This systematic review and meta‐analysis provide, to our knowledge, the first and most comprehensive head‐to‐head synthesis directly comparing CS and DP in PF. Unlike previous meta‐analyses, such as Lai et al. [19], which included only two RCTs for this comparison, our analysis incorporated seven studies (five RCTs) and explicitly separated VAS domains (general, morning, walking) while including functional and structural outcomes. This allowed us to capture more granular differences between the two modalities. Pooled analyses revealed that CS was associated with significantly greater short‐term improvements in pain (across all VAS domains) and Foot Function Index (FFI), as well as reductions in PFT, indicating superior early relief. However, by 3 months, DP showed greater improvement in the FFI, with no significant between‐group differences in the VAS score or PFT. Adverse events were infrequently reported, although one trial noted heel fat pad atrophy with CS and transient paresthesia with DP. These results suggest that while CS is preferable for rapid symptom control, DP may be a better option for maintaining functional recovery, particularly when performed under ultrasound guidance.

### 4.1 Explanation of the Study Findings

PF‐related heel pain is typically unilateral, described as aching, stabbing, or burning, worsened by prolonged standing, walking, or high‐impact exercise, and transiently relieved by rest or gradual movement [32]. In the studies included in our analysis, pain severity was measured via the VAS score, and pooled results demonstrated that CS injections produced a significant short‐term reduction in pain. These findings are consistent with those of previous meta‐analyses, including those by Lai et al. [19], who reported that DP was less effective than CS during early follow‐up, and by Chen et al. [33], who reported that CS yielded superior short‐term pain reduction compared with noninvasive treatments such as physical therapy. Similarly, Kumar et al. [34] observed greater pain improvement at 1.5 months with CS than with platelet‐rich plasma (PRP) injections. The early analgesic effect of CS is attributed to its potent anti‐inflammatory action, which involves the inhibition of phospholipase A_2_ and subsequent suppression of prostaglandin and leukotriene synthesis, thereby reducing local inflammatory signaling in the plantar fascia [35]. However, because this mechanism does not address the underlying degenerative pathology, it fails to promote structural repair [36]. Consequently, the clinical benefits often wane within weeks to months, with no advantage in terms of plantar fascia thickness or long‐term pain relief compared with other modalities [37]. Repeated injections, sometimes administered at or beyond 12 weeks [37, 38], increase cumulative exposure and the associated risk of complications such as fat pad atrophy, calcaneal osteomyelitis, abscess formation, and plantar fascia rupture [39, 40, 41, 42].

The FFI, a validated patient‐reported outcome measure evaluating pain, disability, and activity limitation, is frequently employed in PF trials to quantify functional recovery. In our meta‐analysis, compared with DP, CS significantly improved FFI scores at short‐term follow‐up (0.5–1 month), indicating faster early functional gains. This finding is consistent with Lai et al.’s [19] and Herber et al.’s [18] observations, although their conclusions were based on far fewer comparative trials. Additionally, individual clinical trials have documented marked improvements in function and pain within the first 1–3 months after CS administration, although these benefits typically diminish by 6 months [37, 43].

Although CS appears to accelerate short‐term restoration of foot function, our 3‐month pooled analysis—incorporating five studies—revealed a reversal of this trend, with DP outperforming CS in terms of FFI improvement. This pattern mirrors findings from other regenerative therapy studies, such as Zuo et al.’s [3] PRP versus CS meta‐analysis, which demonstrated that regenerative treatments surpassed CS after the early postinjection window. In contrast, Ye et al. [44] reported no significant functional difference between CS and PRP at 3 months. This discrepancy may reflect methodological differences: Zuo et al. [3] included a larger number of trials, broader functional outcome measures, and grouped follow‐up into defined mid‐term intervals (3–6 months), whereas Ye et al.’s smaller dataset, variation in functional scales, and potentially more heterogeneous follow‐up timing may have reduced the statistical power to detect differences. Such variability underscores the importance of consistent outcome measurement and follow‐up definitions when interpreting comparative efficacy. Additional evidence from the literature supports our findings: In the study by Pérez et al. [2], CS achieved less FFI improvement than did extracorporeal shockwave therapy (ESWT) at 3 months. Studies evaluating DP have consistently demonstrated meaningful functional gains, outperforming placebo and achieving efficacy comparable to that of PRP [19, 44]. Although PRP may facilitate faster early functional recovery, DP appears to offer more sustained benefits over time [45, 46]. In contrast, the initial advantage of CS in terms of functional improvement often diminishes, rendering it generally less effective than DP at longer follow‐up. These findings align with our pooled results and further reinforce the role of DP as a preferable option when long‐term functional restoration is the primary treatment goal.

Building on this observation, it is important to consider the underlying biological rationale for the delayed yet sustained clinical effects of DP. In recent years, numerous studies have evaluated the efficacy of DP in PF [20, 44, 45, 46, 47], as well as in other chronic tendinopathies [48]. DP is a regenerative injection therapy that involves local administration of a hypertonic dextrose solution, which is cost‐effective, widely available, and has a favorable safety profile. Local injection of hypertonic dextrose initiates a mild, targeted inflammatory response, stimulating the release of growth factors and promoting fibroblast proliferation, collagen synthesis, and extracellular matrix remodeling [12, 48, 49, 50]. Animal and human studies have demonstrated connective tissue proliferation and even cartilage regeneration post‐dextrose injection [51, 52, 53, 54]. These biological effects likely underlie the sustained mid‐term gains of DPs observed in our meta‐analysis. Notably, unlike Lai et al. [19], we found a statistically significant 3‐month advantage for DP in function, which may reflect the larger dataset, inclusion of more diverse populations, and standardized pooling of outcomes.

With respect to PFT, our analysis revealed that CS injections achieved greater short‐term reductions than DP injections did, likely due to the suppression of inflammatory edema. This finding aligns with Vatsa et al. [55], who reported significant short‐term improvements in both pain scores and PFT following CS compared with placebo. Similarly, Moneim et al. [56] observed that reductions in PFT correlated with pain relief in the CS group, indicating that even modest structural changes may contribute to symptom improvement. However, other reviews have reported minimal or no structural benefit from CS; for example, Zuo et al. [3] reported no significant PFT change compared with PRP, and Peña‐Martínez et al. [37] reported no advantage over active controls. For DP, several studies have demonstrated its capacity to reduce PFT: Mansiz‐Kaplan et al. [46] reported significant decreases at 7 and 15 weeks, Ahadi et al.’s [20] meta‐analysis revealed a large short‐term effect size, and Mahato et al. [57] and Neelam et al. [32] documented meaningful ultrasonographic improvements alongside pain and functional gains. Although these results suggest that both modalities can induce structural changes, the evidence remains inconsistent, and the correlation between PFT reduction and clinical recovery is not absolute. Therefore, PFT should be interpreted alongside validated pain and function measures to guide treatment decisions.

Our subgroup analysis revealed a clear interaction between injection guidance and treatment efficacy: in the ultrasound‐guided subgroup, DP outperformed CS, whereas in the palpation‐guided subgroup, CS was more effective than DP. This likely reflects differences in therapeutic mechanisms—DP's regenerative effect requires precise delivery into the pathological fascia to trigger a localized healing cascade, making it highly dependent on the targeting accuracy provided by ultrasound, whereas CS's potent anti‐inflammatory action can diffuse more broadly and remain effective even with less precise palpation‐guided injection. This interpretation is supported by prior evidence from Doan et al. [58], who reported that ultrasound‐guided CS achieved greater improvements in plantar fascia thickness and tenderness thresholds than did blind CS but that there was no significant difference in VAS pain scores between guidance techniques. This aligns with our results, where the CS retained pain‐relieving efficacy in the palpation‐guided group, whereas the performance of the DP decreased when ultrasound was not used. The importance of precise localization for DP is further reinforced by previous studies demonstrating that ultrasound‐guided prolotherapy significantly improves outcomes in various musculoskeletal disorders—including plantar fasciitis, temporomandibular joint dysfunction, and lateral epicondylitis—where the accurate deposition of the proliferant is critical to stimulate local healing [14, 58, 59, 60].

Our findings have practical implications for treatment selection. On the basis of current evidence and in line with available clinical guidelines, CS injections may be favored when immediate pain relief is the priority—for example, in athletes requiring rapid return to activity or in patients with severe morning pain impacting daily function. Conversely, DP may be preferred in patients where long‐term functional recovery is prioritized, in those at greater risk for CS‐related adverse effects, or where prior CS injections have failed to produce durable benefits. The choice may also be influenced by procedural factors: our subgroup analysis revealed that ultrasound‐guided injections favored DP, whereas palpation‐guided methods yielded better outcomes for CS. This likely reflects the DP's dependence on precise delivery to the pathological site, whereas the effect of the CS can diffuse more broadly even with less targeted placement.

Significant heterogeneity was identified across various outcomes in our analysis, including pain and functional measures. Although subgroup analyses and sensitivity testing reduced some of this variability, residual heterogeneity likely stemmed from methodological differences such as study design (RCTs vs. cohort studies), injection guidance technique (ultrasound vs. palpation), and variation in injectate composition and dosing schedules. For the DP groups, the concentrations ranged from 5% to 50%, with considerable variability in the number of sessions and treatment intervals. These protocol differences appeared to influence short‐term foot function outcome (FFI). For example, Karakılıç et al. conducted two ultrasound‐guided sessions of 30% DP at 2‐week intervals, achieving favorable short‐term improvements comparable to those of corticosteroid injection. Mousa et al., using a similar concentration (25%) but palpation‐guided delivery over multiple sessions, also reported significant early functional gains, surpassing results from some studies with lower concentrations or fewer sessions. Conversely, Teymouri et al. administered a single ultrasound‐guided injection of high‐concentration (50%) DP but observed smaller short‐term functional improvements, suggesting that repeated dosing may be more beneficial than a single high‐dose administration. The lowest gains were reported by Uğurlar et al., who used three ultrasound‐guided sessions of 5% dextrose, underscoring the likely role of concentration in treatment efficacy. Collectively, these differences in intervention protocols, along with the variability in corticosteroid formulation and adjunctive anesthetic use, are likely contributors to the heterogeneity observed in our pooled estimates.

### 4.2 Strengths and Limitations

This review has several strengths. This is the first head‐to‐head meta‐analysis to compare DP and CS in PF using domain‐specific VAS scores and standardized outcome scales. We also explored injection guidance as a potential effect modifier, which provides valuable clinical insights.

However, several limitations need to be considered: Firstly, the relatively small number of included studies (seven in total) limited our ability to conduct sufficient subgroup analyses to achieve conclusive results. Secondly, variability in study design, as both RCTs and cohort studies were included, could have introduced potential bias; we addressed this by performing risk‐of‐bias assessments for all studies and conducting sensitivity analyses to evaluate the impact of study design on our findings. Thirdly, injectate type and concentration, as well as outcome definitions, varied across studies, which might have influenced the pooled results. Fourthly, adverse events were inconsistently reported, preventing robust safety conclusions. Fifthly, high *I* ^2^ values in some analyses and the limited number of studies restrict the generalizability of results and precluded formal assessment of publication bias. Lastly, the lack of long‐term follow‐up (beyond 3 months) in most studies limits our ability to assess the durability of treatment effects.

### 4.3 Recommendation

Future research should prioritize the conduct of large, blinded randomized controlled trials with adequate sample sizes and long‐term follow‐up durations of at least 6–12 months. Studies should stratify participants by the injection guidance method and ensure standardized injectate concentration, volume, and dosing frequency. Core outcome sets should be adopted, encompassing pain (with separation by domain: general, morning, walking), function (FFI, FAAM), structure (PFT, echogenicity), and safety (adverse events, fat pad thickness). Furthermore, the inclusion of patient‐reported measures such as the SF‐36 score, heel sensitivity index, and walking distance, as well as functional indices such as the American Foot and Ankle Scale (AFAS), would increase the clinical relevance of future findings. Importantly, cost‐effectiveness analyses comparing ultrasound‐guided DP and CS would help guide therapeutic decision‐making in real‐world settings.

## 5 Conclusion

This meta‐analysis revealed that CS injections yield superior short‐term improvements in pain and function, whereas DPs provide greater mid‐term Functional Gain Index (FFI) and achieve superior general pain reduction when ultrasound guidance is used. Both treatments demonstrate comparable pain relief (morning and walking VAS) and plantar fascia thickness reduction by 3 months, with low adverse event rates. Future high‐quality trials with standardized injection protocols—including concentration, type, and frequency—and extended follow‐up are warranted to clarify long‐term efficacy and optimize treatment selection.

## Author Contributions

**Ruaa Mustafa Qafesha:** conceptualization, methodology, data curation, formal analysis, visualization, writing – original draft, writing – review and editing. **Hammam Anas Ishreiteh:** conceptualization, data curation, writing – original draft, writing – review and editing. **Adli Luay Nassourah:** conceptualization, data curation, writing – original draft, writing – review and editing. **Omar Islam Tawil:** conceptualization, visualization, writing – original draft, writing – review and editing. **Doaa Mashaly:** conceptualization, data curation, writing – original draft, writing – review and editing.

## Funding

The authors have nothing to report.

## Ethics Statement

The study was performed in accordance with the ethical standards of the 1964 Declaration of Helsinki and its later amendments. The manuscript does not contain clinical studies or patient data.

## Consent

The authors have nothing to report.

## Conflicts of Interest

The authors declare no conflicts of interest.

## Supporting information


Supporting Information S1

## Data Availability

The authors confirm that the data supporting the findings of this study are available within the article and its supplementary material. Raw data that support the findings of this study are available from the corresponding author upon reasonable request.
