# Supplementary material for: Efficacy and Safety of Dextrose Prolotherapy Versus Corticosteroid Injections in Plantar Fasciitis: A Systematic Review and Meta‐Analysis
Source: J Foot Ankle Res. 2026 Feb 17;19(1):e70135. doi: 10.1002/jfa2.70135 (PMC12913222; doi:10.1002/jfa2.70135)
Supplement: Supplementary file 1 — Supporting Information S1 [file JFA2-19-e70135-s001.docx]

Efficacy and Safety of Dextrose Prolotherapy versus Corticosteroid Injections in Plantar Fasciitis: A Systematic Review and Meta-Analysis

**Supplementary File**

| **Table S1: Search strategy used across databases** | | | |
| --- | --- | --- | --- |
| **Databases** | **Search Strategy** | **N** |  |
| **PubMed** | ((Plantar Fasciitis OR plantar fasciopathy OR plantar fasciosis OR plantar heel pain OR heel pain) AND (prolotherapy OR dextrose prolotherapy OR hypertonic dextrose OR dextrose injection OR glucose injection OR dextrose OR glucose) AND (corticosteroid OR steroid OR glucocorticoid OR triamcinolone OR prednisolone OR prednisone OR methylprednisolone)) | 22 |  |
| **Google Scholar** | ("Plantar Fasciitis" OR "plantar fasciopathy" OR "plantar fasciosis" OR "plantar heel pain") **AND** ("prolotherapy" OR "dextrose prolotherapy" OR "hypertonic dextrose" OR "dextrose injection" OR "glucose injection" OR "dextrose" OR "glucose") **AND** ("corticosteroid" OR "steroid" OR "glucocorticoid" OR "triamcinolone" OR "prednisolone" OR "prednisone" OR "methylprednisolone") -"case report" -review -protocol -editorial -animal | 128 |  |
| **Scopus** | (**TITLE-ABS-KEY** ("plantar fasciitis" OR "plantar heel pain" OR "plantar fasciopathy" OR "plantar fasciosis" OR "heel pain") AND **TITLE-ABS-KEY** ("prolotherapy" OR "dextrose" OR "glucose" OR "glucose injection" OR "hyperosmolar dextrose") AND **TITLE-ABS-KEY** ("corticosteroid" OR "steroid" OR "methylprednisolone" OR prednisone OR "triamcinolone" OR "prednisolone")) | 55 |  |
| **WOS** | **TS =** (plantar fasciitis OR plantar heel pain OR plantar fasciopathy OR plantar fasciosis OR "heel pain") **AND** **TS =** (prolotherapy OR dextrose OR glucose injection OR glucose OR hyperosmolar dextrose) **AND** **TS =** (corticosteroid OR steroid OR methylprednisolone OR prednisone OR triamcinolone OR prednisolone) | 28 |  |
| **Cochrane** | **ti,ab,kw** (plantar fasciitis OR plantar heel pain OR plantar fasciopathy OR plantar fasciosis OR heel pain) **AND** (prolotherapy OR dextrose OR glucose OR glucose injection OR hyperosmolar dextrose OR dextrose OR glucose)**:ti,ab,kw** **AND** (corticosteroid OR steroid OR prednisone OR methylprednisolone OR triamcinolone OR prednisolone)**:ti,ab,kw** | 25 |  |

| **Table S2: Records Excluded from Systematic Review and Meta-Analysis in Full Text Screening Phase.** | | |
| --- | --- | --- |
| **Reference** | | **Reason of exclusion** |
| 1. **1.** | *Comparative effectiveness of extracorporeal shock wave therapy, local corticosteroid injection and dextrose prolotherapy in treatment of chronic plantar fasciitis: a randomized controlled study. PACTR202504633378954; 2025. Available from:* <https://trialsearch.who.int/Trial2.aspx?TrialID=PACTR202504633378954> | Duplicate |
| 1. **2.** | *Karakiliç GD, Aras M, Büyük F, Bakirci ES. Prolotherapy versus phonophoresis and corticosteroid injections for the treatment of plantar fasciitis: a randomized, double-blind clinical trial. J Foot Ankle Surg. 2023;62(6):922-927. doi:10.1053/j.jfas.2023.04.010* | Duplicate |
| 1. **3.** | *Cochrane Central Register of Controlled Trials. Prolotherapy and corticosteroid injection in plantar fasciitis. 2021; Issue 07. Available from:* <https://www.cochranelibrary.com/central/doi/10.1002/central/CN-02282344/full> | Duplicate |
| 1. **4.** | *Cochrane Central Register of Controlled Trials. Effects of dextrose and corticosteroid on plantar fasciitis treatment. 2019; Issue 3. Available from:* [*https://www.cochranelibrary.com/central/doi/10.1002/central/CN-01849412/full*](https://www.cochranelibrary.com/central/doi/10.1002/central/CN-01849412/full) | Duplicate |
| 1. **6.** | Moneim NHA, Hemed MA, ten Klooster PM, Rasker JJ, El Shaarawy NK. Chronic plantar fasciitis treatment: a randomized trial comparing corticosteroid injections followed by therapeutic ultrasound with extracorporeal shock wave therapy. *Rheumato.* 2023;3(3):12. doi:10.3390/rheumato3030012 | Wrong Comparator |
| 1. **7.** | Mansiz-Kaplan B, Nacir B, Pervane-Vural S, Duyur-Cakit B, Genc H. *Effect of dextrose prolotherapy on pain intensity, disability, and plantar fascia thickness in unilateral plantar fasciitis: a randomized, controlled, double-blind study.* Am J Phys Med Rehabil. 2020;99(4):318-324. doi:10.1097/PHM.0000000000001330 | Wrong Comparator |
| 1. **8.** | Lee DO, Yoo JH, Cho HI, Cho S, Cho HR. *Comparing effectiveness of polydeoxyribonucleotide injection and corticosteroid injection in plantar fasciitis treatment: a prospective randomized clinical study.* Foot Ankle Surg. 2020;26(6):657-661. doi:10.1016/j.fas.2019.08.005 | Wrong Comparator |
| 1. **9.** | *Mahindra P, Yamin M, Selhi HS, Singla S, Soni A.* Chronic plantar fasciitis: effect of platelet-rich plasma, corticosteroid, and placebo. *Orthopedics. 2016;39(2):e285–e289. doi:10.3928/01477447-20160222-01* | Wrong Comparator |
| 1. **10.** | *Melda Y, Eroğlu A.* Comparison of the effects of stretching exercises, prolotherapy, ESWT and corticosteroid injection on VAS scores in inactive adults with chronic plantar fasciitis: a retrospective cohort study. *Acta Med Mediterr. 2022;38(4):2357–2362. doi:10.19193/0393-6384_2022_4_359* | Full Text Unavailable |
| 1. **11.** | *Cochrane Central Register of Controlled Trials.* Ultrasound-guided prolotherapy, oxygen-ozone and corticosteroid injection for the treatment of plantar fasciitis. *2020; Issue 11. Available from:* [*https://www.cochranelibrary.com/central/doi/10.1002/central/CN-02187309/full*](https://www.cochranelibrary.com/central/doi/10.1002/central/CN-02187309/full) | Full Text Unavailable |
| 1. **12.** | *Cochrane Central Register of Controlled Trials.* Efficacy of dextrose 25% injection vs. steroid injection in the treatment of heel spur. *2011; Issue 3. Available from:*<https://www.cochranelibrary.com/central/doi/10.1002/central/CN-01879105/full> | Full Text Unavailable |
| 1. **13.** | Effect of Extracorporeal Shockwave Therapy on Gait Parameters in Patients With Plantar Fasciitis. *Cochrane Central Register of Controlled Trials* (CENTRAL). 2024, Issue 4. Record ID: NCT06310122. Available from: <https://clinicaltrials.gov/ct2/show/NCT06310122> | Wrong Intervention |
| 1. **14.** | Dev K, Meena AS, Meena M, Joshi M. A randomized control study comparing standard care vs ultrasonography-guided single-dose methylprednisolone acetate for plantar fasciopathy. *Eur J Mol Clin Med.* 2022. | Wrong Intervention |
| 1. **5.** | *Demir G, Okumus M, Karagoz A, Kultur T. Prolotherapy versus corticosteroid injections and phonophoresis for the treatment of plantar fasciitis: a randomized controlled trial. Presented at: 2015 ACR/ARHP Annual Meeting; 2015 Sep 29; San Francisco, CA. Abstract 1410. Available from:* <https://acrabstracts.org/abstract/prolotherapy-versus-corticosteroid-injections-and-phonophoresis-for-the-treatment-of-plantar-fasciitis-a-randomized-controlled-trial/> | Abstract |
| 1. **15.** | El-helw MR, Fathallah MM, El Zohjery AK, Mostafa NH. Comparison between the effect of steroid injection, prolotherapy local injection and extracorporeal shock wave therapy in treatment of symptomatic calcaneal spur. *QJM: An International Journal of Medicine*. 2024;117(Supplement_1):hcae070.535. doi:10.1093/qjmed/hcae070.535. | Abstract |
| 1. **16.** | Lone AH, Khursheed O, Rashid S, Mir BA, Nazee FA. Management of chronic plantar fasciitis using hyperosmolar dextrose injection. *J Med Sci Clin Res*. 2015;3(1):3931-3935. | Single Arm |
| 1. **17.** | Mahato A, Gupta MK, Panthee R, Pathak MR. Effectiveness of dextrose prolotherapy in chronic plantar fasciitis: a cross-sectional study. *Birat J Health Sci*. 2023;8(1):1–6. Published 2023-08-21. | Single Arm |
| 1. **18.** | Fullerton BD, Reeves KD. Ultrasonography in regenerative injection (prolotherapy) using dextrose, platelet-rich plasma, and other injectants. *Phys Med Rehabil Clin N Am*. 2010;21(3):585–605. | Review Article |
| 1. **19.** | *Cochrane Central Register of Controlled Trials.*  *An interventional study to compare the effect of steroid injection versus dextrose prolotherapy in management of medial heel pain.* Cochrane Central Register of Controlled Trials*. 2024; Issue 6. Trial ID: CTRI/2024/04/066454. Available from:*<https://trialsearch.who.int/Trial2.aspx?TrialID=CTRI/2024/04/066454> | Ongoing Trial |

| **Table S3. Methodological quality assessment of the included 2 observational studies, based on the NOS for assessing the quality of observational studies.** | | | | | | | | | |
| --- | --- | --- | --- | --- | --- | --- | --- | --- | --- |
| **Study ID** | **Selection** | | | | **Comparability** | **Exposure** | | | **Total score** |
|  | Representativeness of the exposed cohort | Selection of the non-exposed cohort | Ascertainment of exposure | Demonstration that outcome of interest was not present at the start of the study | Comparability of cohorts on the basis of the design or analysis | Assessment of outcome | Was follow-up long enough for outcomes to occur | Adequacy of follow-up of cohorts |  |
| **Puranik 2025** |  | ⋆ | ⋆ | ⋆ | ⋆ | ⋆ | ⋆ | ⋆ | **7** |
| **Calısal 2022** |  | ⋆ | ⋆ | ⋆ | ⋆⋆ | ⋆ | ⋆ |  | **7** |


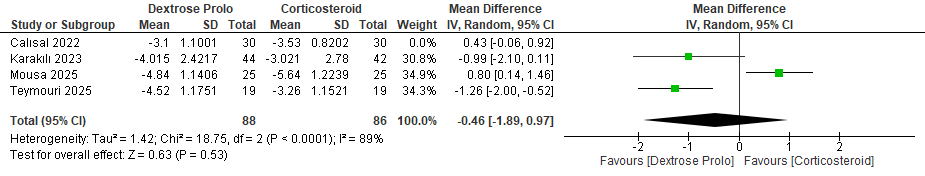


**Fig S1:** Forest plot for comparison between CS and DP regarding general VAS score change at 3 months **after** excluding cohort study (Calısal 2022) from the analysis (RCT only model)


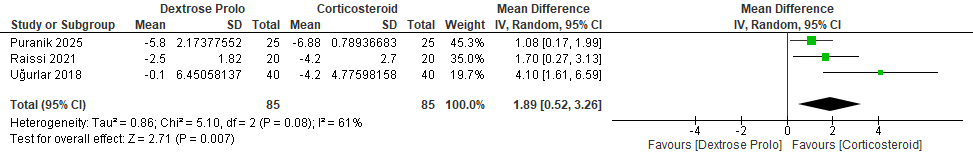


**Fig S2:** Forest plot for comparison between CS and DP regarding Morning VAS score change at 0.5–1 month **before** sensitivity analysis


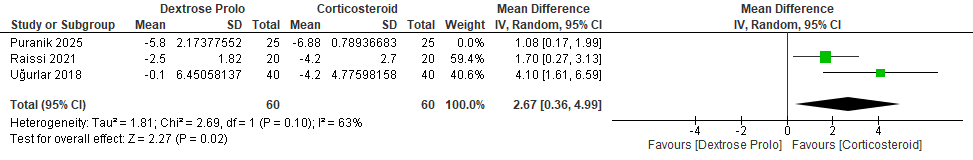


**Fig S3:** Forest plot for comparison between CS and DP regarding morning VAS score change at 0.5–1 month **after** excluding cohort study (puranik 2025) from the analysis (RCT only model)


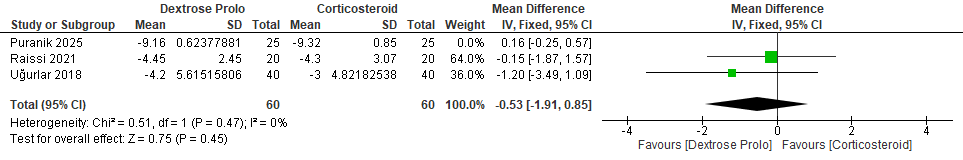


**Fig S4:** Forest plot for comparison between CS and DP regarding morning VAS score change at 3 months **after** excluding cohort study (puranik 2025) from the analysis (RCT only model)


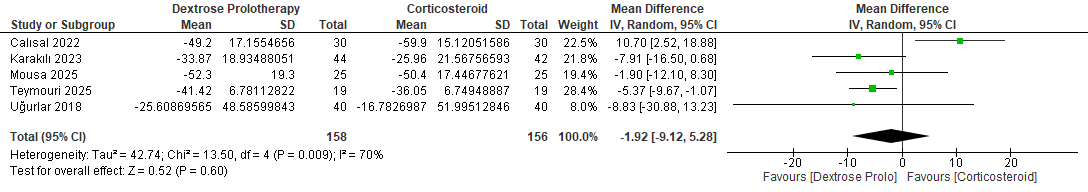


**Fig S5:** Forest plot for comparison between CS and DP regarding Foot Function Index change at 3 months **before** sensitivity analysis.


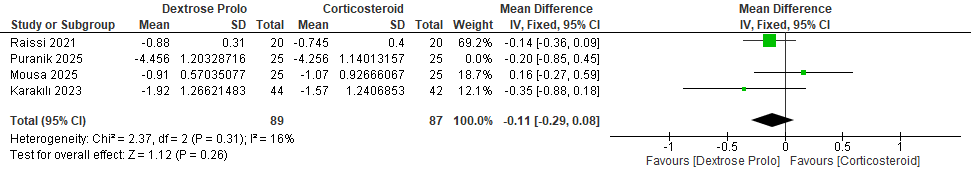


**Fig S6:** Forest plot for comparison between CS and DP regarding plantar fascia thickness change at 3 months **after** excluding cohort study (Puranik 2025) from the analysis (RCT only model).

.
